# Assessment of the Interdependencies Between High-Speed Videoendoscopy and Simultaneously Recorded Audio Data in Various Glottal Pathologies

**DOI:** 10.3390/biomedicines13020511

**Published:** 2025-02-18

**Authors:** Magdalena M. Pietrzak, Wioletta Pietruszewska, Magda Barańska, Aleksander Rycerz, Konrad Stawiski, Ewa Niebudek-Bogusz

**Affiliations:** 1 Department of Otolaryngology, Head and Neck Oncology, Medical University of Lodz, 90-419 Lodz, Poland; magdalena.marianna.kowalczyk@gmail.com (M.M.P.);; 2 Department of Biostatistics and Translational Medicine, Medical University of Lodz, 90-419 Lodz, Poland

**Keywords:** larynx, high-speed videolaryngoscopy, acoustic analysis, parameters, kymography, glottal pathology

## Abstract

**Background**: This study aimed to investigate the relationships between kymographic parameters derived from high-speed videoendoscopy (HSV) and simultaneously recorded acoustic signals. The research provides insights into the vibratory dynamics of various glottal pathologies, assessed across different glottal widths, and their mutual relations with audio data. **Methods**: The study included 192 participants categorized as normophonic or having functional or organic lesions (benign, premalignant, and malignant). Parameters describing vocal fold oscillations were calculated using HSV kymography for three glottal widths, along with corresponding acoustic data. Initially, linear correlations between these parameters were assessed. Next, the consistency in cycle detection and its influence on the correlation levels were evaluated. **Results**: The fundamental frequency (F0) and mean Jitter (Jita) showed the highest correlations between the HSV- and audio-determined parameters (F0: 0.97, Jita: 0.40–0.70), with even stronger correlations when the number of detected cycles was consistent (F0: 0.99, Jita: 0.68–0.98). The correlations for other parameters ranged from low to moderate, with no significant differences observed between the diagnostic subgroups (functional changes and benign and malignant glottal lesions). However, in the premalignant lesions group, high correlations (0.77–0.9) were observed between the HSV and audio parameters, but only for measures describing period perturbations. Beyond F0 and mean Jitter, consistency in cycle detection did not significantly affect correlation levels. **Conclusions**: The simultaneous audio signal proved useful in verifying the accuracy of HSV quantification measures, particularly for F0, which showed strong agreement between the methods. Discrepancies in other parameters and low correlations between HSV-derived kymography and audio data may suggest the influence of the throat, mouth, and nose resonators, which are added to the glottal signal. While the kymographic analysis based on HSV provides detailed descriptions of vocal fold oscillations, it does not fully capture the three-dimensional structure and complex functionality of the vocal folds.

## 1. Introduction

Over the years, various methods have been developed for laryngeal examination. While white-light endoscopy is widely used today, advancements in visualization techniques have introduced new tools for assessing the larynx. Some of these methods focus on the functional evaluation of vocal fold oscillations, such as laryngovideostroboscopy (LVS), while others aim to enhance the detection of lesions that are difficult to identify under white light, such as narrow band imaging (NBI) [1]. LVS remains the gold standard for assessing vocal fold oscillatory function; however, it is limited in cases of non-periodic voice disorders or when patients struggle with prolonged phonation. High-speed videoendoscopy (HSV) offers an alternative for evaluating vocal fold vibratory patterns. With its high spatial resolution and sampling rate, HSV enables detailed observation of individual cycles in both normal and pathological states of the glottis. Despite the documented benefits of HSV, there is still a need for objective parameters to establish its superiority over LVS [2,3,4]. Commonly used methods for HSV analysis include visual techniques, e.g., phonovibrography (PVG) or laryngotopography [5]. Other techniques for processing sequentially acquired images results in objective parameterization, e.g., kymography, the adaptive thresholding approach, or the snake-based fitting approach (SBFA) [6]. Changes in the glottal function could be described as Glottal Area Waveform (GAW), describing the changing area of the glottal gap in time, or Glottal Width Waveform (GWW), describing the glottal widths in time. Research suggests that the degree of oscillation varies along the vocal fold, with the greatest oscillatory activity occurring in the middle segment. Consequently, some authors recommend focusing kymographic analysis on the midsection of the glottis [7,8]. While endoscopic laryngeal examinations provide direct data about the glottis, acoustic analyses capture subharmonics from the supraglottal vocal tract and changes made by the influence of throat, mouth, and nose resonators, which are added to the signal generated in the larynx [9]. While it seemed to be reasonable to hypothesize a relationship between the acoustic signal and vocal fold vibratory patterns, as they came from the same source, the glottis, other influences on the acoustic data are undeniable.

Moreover, when endoscopic imaging and acoustic analysis are performed as separate methods, they capture data from different phonatory patterns, complicating reliable comparisons—even when similar parameters are assessed. Some systems enable simultaneous recording of HSV and audio data, which has been proposed for verifying the accuracy of HSV examinations and analyses or for guiding the segmentation of HSV recordings [10,11]. Other studies have used simultaneous audio recordings to provide additional insights into vocal fold oscillations and to enhance the understanding of vocal physiology [12]. However, few studies have examined the relationships between HSV and audio-based parameters. Most research has focused on assessing parameter values and their changes following interventions [13].

It is important to distinguish between absolute agreement and functional interrelationships between HSV and acoustic measures. Absolute agreement implies identical measurements across both modalities under ideal conditions; however, due to physiological factors such as vocal tract resonances and methodological differences in data acquisition, perfect agreement is unlikely. Instead, the study aims to explore functional interrelationships—identifying which parameters consistently correlate despite these influencing factors and which ones require additional contextual interpretation.

Furthermore, the integration of simultaneous HSV and audio recordings offers an opportunity to validate clinical assessments rather than achieving identical measurements. By identifying consistent trends across both modalities, clinicians can gain complementary insights into vocal fold dynamics, ensuring more accurate diagnosis and treatment planning. This combined approach has the potential to reduce subjective variability in vocal fold assessments and enhance confidence in clinical decision making.

This study aims to analyze the correlations between HSV kymographic parameters by characterizing the periodicity and amplitude of vocal fold oscillations and acoustic parameters derived from simultaneously recorded audio signals. The mutual relations were assessed for all the study subjects and for the subgroups of patients categorized by the following diagnosis: normophonic and with functional and organic voice disorders. By exploring these relationships, this study seeks to enhance the diagnostic utility of HSV and acoustic analyses, particularly for complex glottal pathologies where a comprehensive understanding of vibratory dynamics is crucial. Furthermore, this study examines the impact of variability in cycle detection on the correlation levels, aiming to assess the potential influence of software artifacts on the results.

## 2. Materials and Methods

### 2.1. Study Group

In this study, there were 283 subjects hospitalized at the Department of Otolaryngology, Head and Neck Oncology, Medical University of Lodz, Poland, and a total number of 192 were proceeded after evaluation of the recordings’ quality for further analysis. The quality of the recordings was assessed by two experienced ENT/phoniatricans. HSV data were included in the study when a kymographic analysis was possible (visible glottal opening, visible about 85% of the glottal length, and both vocal folds visible). Audio recordings were then assessed on a three-point scale: 0—good quality, no background noise; 1—slight background noise but quality good enough for the analysis; and 2—analysis impossible due to background noise. In this study, we included patients rated as “0” and “1”.

Out of the 283 participants initially recruited for this study, 91 were excluded based on predefined criteria to ensure the quality and reliability of the data used for analysis. Specifically, recordings were excluded if the glottis was not sufficiently visible for kymographic analysis (e.g., visibility of less than 85% of the glottal length), if both vocal folds were not clearly visible, or if there was significant background noise in the audio recordings (marked as “2” in the abovementioned criteria). The exclusion breakdown included 13 cases due to poor glottal visibility, 48 cases with inadequate audio quality, and 30 cases because less than 10 cycles were detected for the analysis. These criteria were established to maintain a high standard of data quality, ensuring the validity of parameter calculations and subsequent statistical analyses.

The group included in the study was divided into subgroups: 36 normophonic cases (18.7%), 41 functional disorders (21.4%), and 115 organic lesions (59.9%). In the group of organic lesions, all patients underwent a biopsy, and a histopathological result was obtained. Most of them presented with benign lesions, 71 (61.7%), mainly with Reinke’s oedema and polyps. The group of patients described as other were mostly functional changes and a few cases of inflammatory lesions of vocal folds. Group characteristics according to the clinical and histopathological examination are presented in Table 1. In the literature, patients are usually divided according to gender for analysis due to differences in voice characteristics. Since we investigated the correlations between parameters for each patient separately and did not compare different patients, we did not divide the group by gender. The mean age in the study group was 57 ± 15.

### 2.2. Equipment and Instrumentation

HSV recordings were carried out using a rigid, oval-shaped endoscope (Xion ϕ10.0, Berlin, Germany) with light input in Storz standard matched to a 4.8 mm fiber optic light cable. The HSV images of the larynx were registered with an Advanced Larynx Imager System (ALIS) manufactured by Diagnova Technologies (Wroclaw, Poland), equipped with an innovative endoscope laser light source (ALIS Lum-MF1) and a laryngeal high-speed camera (ALIS Cam HS-1) with a CMOS image sensor with a color filter array (the classic Bayer filter) and a global shutter (http://diagnova.eu/dist/files/DiagNova_advanced_larynx_imaging_system.pdf, date access 1 January 2025). Images were recorded ranging from 2400 to 3200 frames per second, ranging from 544 × 512 to 416 × 416 pixels correspondingly. The microphone was placed on the collar of the patient’s clothes (10–20 cm from mouth) for the simultaneous recording of the acoustic signal.

### 2.3. Recording Analysis

**Figure 1:**
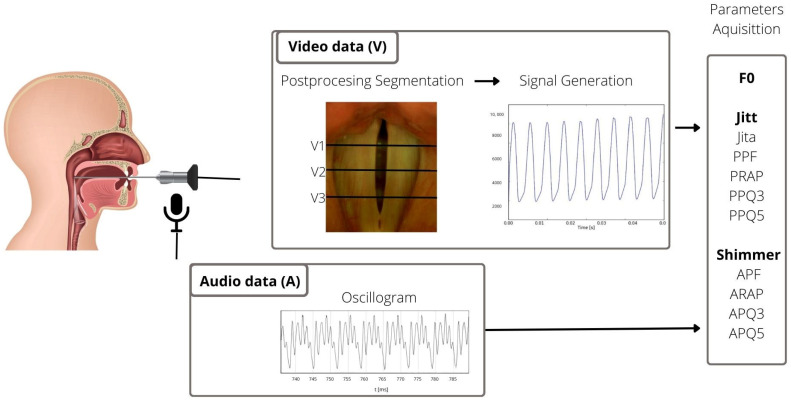
A schematic illustration of the examination process with the rigid endoscope using high-speed videoendoscopy (V) with the simultaneously recorded audio data (A) resulted in the parameter acquisition as listed below: F0, Jitter and its measures (Jita, PPF, PRAP, PPQ3, and PPQ5), and Shimmer and its measures (APF, ARAP, APQ3, and APQ5). The parameters were calculated for three glottal widths, V1—posterior, V2—middle, and V3—anterior, and simultaneously recorded audio data (A).

**Figure 2:**
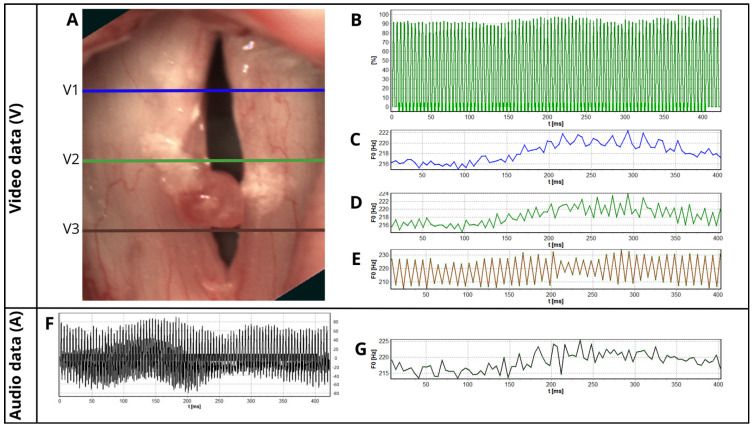
Data obtained during the HSV examination with the simultaneously recorded audio signal—an example of subject with polyp of the right vocal fold. Video data (V): (**A**) image of the glottis with marked glottal widths for the analysis; V1 (blue line)—posterior, V2 (green line)—middle, V3 (brown line)—anterior. (**B**) Middle glottal width waveform V2 (green line) with detected cycles marked as the green pointers in the bottom of the graph. (**C**) Fundamental frequency (F0) in time for the posterior glottal width V1 (blue line). (**D**) Fundamental frequency (F0) in time for the middle glottal width V2 (green line). (**E**) Fundamental frequency (F0) in time for the anterior glottal width V3 (brown line). Audio data (A): (**F**) oscillogram with detected cycles marked as the black pointers. (**G**) Fundamental frequency (F0) in time for the audio data.

For the analysis, part of the recording containing 600 frames from the high-speed video and corresponding audio recording were chosen. The most stable phonation segments were selected to ensure accurate parameter computation. Then, an ENT specialist manually selected three glottal widths for the analysis, anterior (V3), middle (V2), and posterior (V1), about 25%, 50%, and 75% the length of the glottis, respectively (Figure 1) This type of selection allows avoiding the error when the software incorrectly marks the glottal gap, especially when is not fully visible. The cycles for each glottal width and audio signal were detected automatically by the software, and the data about the number of detected cycles were collected in the database as the numerical results and marked on the graph of glottis function in time (Figure 2). For further analysis, only patients with at least 10 cycles detected were included, a number which made it possible to calculate all parameters. Due to this condition, we excluded 30 patients.

### 2.4. Investigated Parameters

Each patient had a calculated set of parameters for the high-speed video based on the glottal width waveform (GWW), which is a graph of glottal width changes in the function of time for three cross-sections of the glottis and audio data. Vocal fold oscillations were described by the period and amplitude perturbation measures calculated based on the formulas and with the usage of dedicated Diagnova software [14].

The analysis included the parameters listed below: fundamental frequency (F0); Jitter and its measures describing the period perturbations, Jita (mean Jitter), the Period Perturbation Factor (PPF), the Period Relative Average Perturbation (PRAP), and the Period Perturbation Quotient with an average of 3 and 5 vocal cycles (PPQ3 and PPQ5); and Shimmer and its measures describing the amplitude perturbations, the Amplitude Perturbation Factor (APF), the Amplitude Relative Average Perturbation (ARAP), and the Amplitude Perturbation Quotient with an average area of 3 and 5 vocal cycles (APQ3 and APQ5). A schematic illustration of the examination process is presented in Figure 1, and an example of the obtained data for further analysis is presented in the Figure 2. More details about HSV instrumentation and postprocessing can be found in the previous publication [15].

### 2.5. Cycle Detection Variability Coefficient

The data regarding the number of detected cycles for each glottal width and audio signal were collected in the database both as numerical data and marked on the graph of glottal function in time (Figure 2B). There are no validated methods for assessing the number of detected cycles that allow for an accurate analysis. Therefore, we considered a cycle count of ten or more as sufficient and calculated the variability coefficient for the number of detected cycles to assess the comparability of the obtained audio and video parameters. Also, we checked if the higher amount of the analyzed cycles (fifty or more) improves the correlations to check if our inclusion criterion (ten cycles or more) is reasonable. To exclude the influence of software error in the cycle detection on the correlations between the glottal widths and audio data, we selected the group with a consistent number of analyzed cycles. The variability coefficient was calculated using the following formula:

$$V=\frac{s}{\bar{x}}$$

where the components are as follows:

*s*—The standard deviation of the number of cycles detected for the video and audio data.

$\bar{x}$—The arithmetic mean of the number of cycles detected for the video and audio data.

This coefficient was calculated for each patient based on the number of cycles in three glottal widths for the high-speed video and audio signal analysis. It is generally accepted that a variability < 25% indicates low diversity. In our study, we examined the distribution of this parameter and adopted a variability value of ≤2% as the threshold for defining the absence of diversity in the number of detected cycles. This threshold represented the second quartile of the distribution of the calculated variability coefficients.

We wanted to investigate whether the number of cycles detected in the three glottal widths differs from the number of cycles detected in the audio signal. Then, we checked whether this difference was related to the variability in measurements of a given parameter between the audio and video analysis. For this purpose, we determined the coefficient defined by the formula

$$CD=\frac{\left|V_{L}-A\right|}{\bar{x}}$$

where the components are as follows:

*V_L_*—The number of cycles from the video measurement value of a given patient at a given glottal width.

*A*—The number of cycles from the audio measurement value for a given patient at a given glottal width.

$\bar{x}$—The arithmetic mean of both measurements concerning the number of cycles for a given patient at a given glottal width.

Then, the Pearson correlation coefficient for those two coefficients described above (V and CD) concerning the number of cycles/parameter values was calculated. The same method was used to calculate the correlations between the values of the parameters calculated based on the three glottal widths and audio data. To assess the consistency of the parameters obtained from the HSV and acoustic recordings, we used the intra-class correlation coefficient (ICC), following the recommendations of Koo and Li (2016) [16]. A two-way mixed-effects model (ICC(3,1)) was applied to evaluate the consistency of the measurements across modalities while accounting for potential systematic differences. We employed a two-way random-effects ICC model to evaluate absolute agreement. This approach was chosen to account for variability in parameter measurements across different glottal widths and audio data, ensuring robust assessment of the consistency and agreement between the two modalities.

A statistical analysis was performed using RStudio 4.2.0; *p*-values < 0.05 were considered statistically significant. The Shapiro–Wilk test was used to check the results for normal distribution of the investigated parameters. All procedures performed in studies involving human participants were in accordance with the ethical standards of the institutional and national research committee and with the 1964 Helsinki Declaration and its later amendments or comparable ethical standards. Informed consent was obtained from all individual participants involved in the study. Approval for this study was granted by the Ethical Committee of the Medical University of Lodz (decision no. RNN/96/20/KE 08/04/2020).

## 3. Results

### 3.1. Correlations Between the Parameter Values Calculated Based on the High-Speed Video and Audio Data

**Figure 3:**
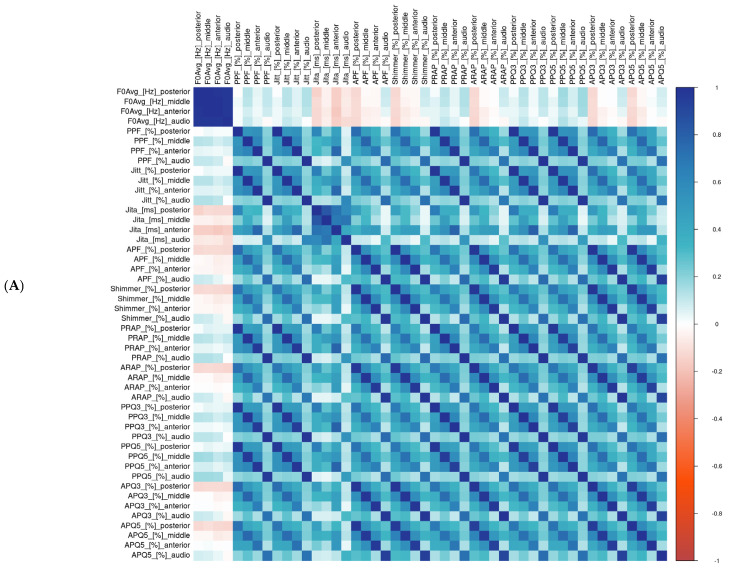

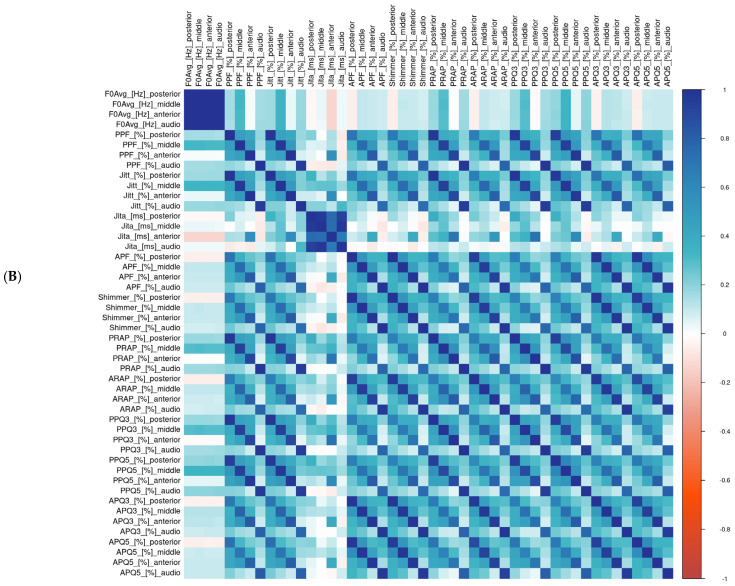
Heat maps showing correlations between kymographic parameters based on the high-speed videoendoscopy (HSV) including different glottal widths (anterior, middle, and posterior) and parameters based on simultaneously recorded audio signals. Heat map scale on the right side of the figure: dark blue, high positive correlation; white, no correlation; dark red, high negative correlation. (**A**) Whole group; (**B**) patients with consistent (≤2% variability) cycle detection for different glottal widths based on the high-speed video (HSV) and audio signals. Database with exact values of correlations and column legends included in the Supplementary Materials (Supplementary Table S5).

The Pearson correlations between the parameter values for different glottal widths based on the HSV and audio data mostly ranged from low to moderate (0.2–0.59). High correlations between parameter values based on the audio data and HSV were noted only for the F0 (all cross-sections 0.97) and mean Jitter (Jita; highest for the middle and posterior glottal widths, 0.6–0.7). For all the parameters, the middle glottal width had the highest correlations with the audio data among the investigated parts of the glottis (Figure 3A). When analyzing the selected cases with a consistent number of detected cycles, the level of correlations between the HSV and audio data obtained similar results as in the whole group. However, in those cases, the posterior cross-section of the glottis had the highest correlation for most of the parameters (PPF, Jita, APF, Shimmer, PRAP, ARAP, PPQ3, PPQ5, APQ3, and APQ5); however, very close values were noted for the middle glottal width. In selected cases with consistent cycle detection, the highest correlations between the HSV and audio data were also noted for the F0 and mean Jitter (Jita; very high correlations for the F0 0.99, Jita 0.93–0.97). Moreover, both the F0 and mean Jitter (Jita) had no correlations with any other parameters, while the other parameters presented the correlations as mentioned below (Figure 3B).

For the HSV data, high correlations between the parameters were observed between the Jitter and its measures describing the periodicity perturbations. The highest correlations were observed for different parameters from the Jitter group between the same glottal widths (anterior–anterior, middle–middle, and posterior–posterior) but were also observed at lower levels between different glottal widths inside the Jitter group. The parameters based on the audio data also had high correlations between the parameter values inside the group of Jitter. Shimmer and its measures describing the amplitude presented comparable results. However, there were no high correlations between the parameter values based on the HSV and simultaneous audio data. The correlations described above are presented in the heat maps (Figure 3).

Across the subgroups, fundamental frequency (F0) consistently showed high correlations for all glottal widths and audio data, ranging from 0.9 to 0.99 across the whole group and subgroups. Similarly, mean Jitter (Jita) exhibited high correlations in patients with benign and premalignant lesions (0.8–0.9), though these correlations were negligible to low (0.1–0.3) in patients with malignant lesions and functional changes. These findings suggest that the presence and type of pathology influence the strength of correlations for certain parameters.

For the correlations between HSV and audio data across other parameters, the results were consistent with those seen in the whole group. Except for F0 and mean Jitter (Jita), the correlations generally ranged from low to moderate. Notably, in the group with premalignant lesions, high correlations (0.7–0.9) were observed for parameters describing period perturbations (PPF, Jitter, Jita, PRAP, PPQ3, and PPQ5) in the middle and posterior glottal widths. Additionally, moderate-to-high correlations (0.59–0.99) were observed between various Jitter-related parameters across all glottal widths in the premalignant subgroup. This was the only subgroup where high correlations between the HSV parameters and audio data were noted beyond F0 and mean Jitter (Jita). This premalignant subgroup was the smallest, comprising only 15 patients, most of whom had leukoplakia affecting one vocal fold (80%).

As observed in the whole group, high correlations (~0.9) were found within the Jitter-related parameters describing period perturbations, particularly between the same glottal widths (anterior–anterior, middle–middle, and posterior–posterior). Moderate correlations were noted between different glottal widths within the Jitter group. Shimmer-related parameters, which describe amplitude perturbations, showed comparable trends.

**Figure 4:**
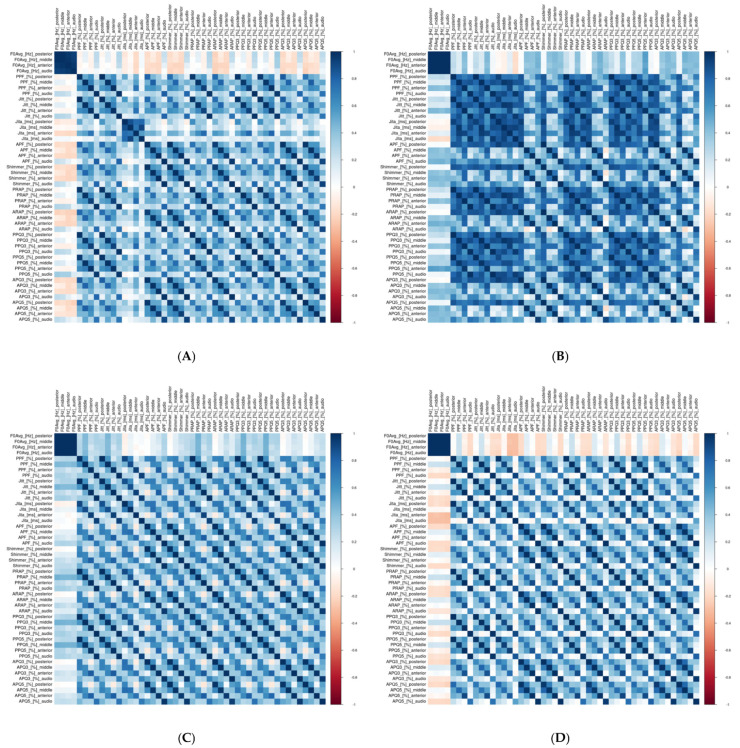
Heat maps showing correlations between kymographic parameters based on the high-speed videoendoscopy (HSV) including different glottal widths (anterior, middle, and posterior) and parameters based on simultaneously recorded audio signals (A) for different groups of patients. Heat map scale on the right side of the figure: dark blue, high positive correlation; white, no correlation; dark red, high negative correlation. (**A**) benign lesions; (**B**) premalignant lesions; (**C**) malignant lesions; (**D**) functional changes. Database with exact values of correlations and column legends included in the Supplementary Materials (Supplementary Tables S1–S4).

The results for each subgroup are visualized in the heat maps in Figure 4. Detailed data on subgroup correlations are available in the Supplementary Materials (Supplementary Tables S1–S5).

### 3.2. Presentation of ICC Results

The ICC analysis revealed excellent agreement for fundamental frequency (F0) across all glottal regions (ICC = 0.88–0.94). Mean Jitter (Jita) also showed good agreement (ICC = 0.73–0.80), particularly in the middle and posterior glottal cross-sections. However, parameters like Shimmer demonstrated only moderate agreement (ICC = 0.58–0.66), indicating the need for careful interpretation (Table 2).

### 3.3. Cycle Detection Variability for the High-Speed Video and Audio Data

In the whole group, 100% cycle detection compliance between the three glottal widths in the HSV and audio signals was noted for 20% of cases. The largest percentage was noted in the normophonic group (30%) and the lowest in the group of benign lesions (15%). However, this percentage is about 50% when we consider the compliance between at least one glottal width and the audio signal. Also, about half of the whole group could be described as compliant in the cycle detection if we set the compliance condition as a variability coefficient ≤ 2% for the cycle detection in the three glottal widths and audio signals.

### 3.4. Cycle Detection Variability Coefficient and the Correlations Between the Parameter Values Calculated Based on the High-Speed Video and Audio Data

**Figure 5:**
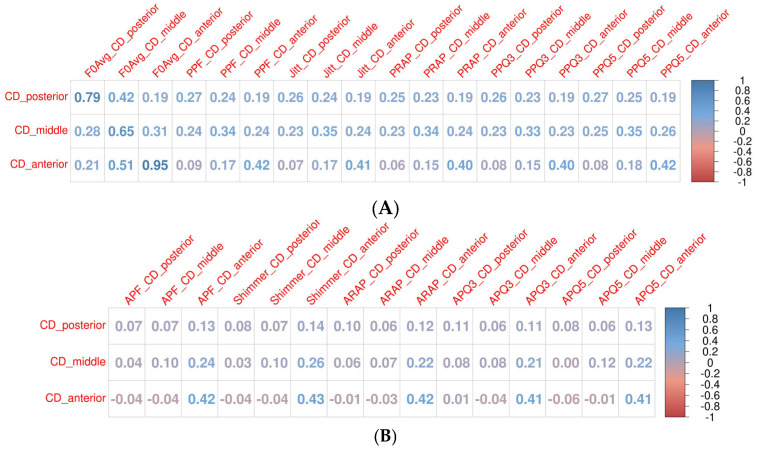
Whole group. Correlations between CD index (on the Y axis) describing the compliance between cycle detection for the high-speed video (HSV) for different glottal widths and audio data (described in detail in the Section 2) and the values of the kymographic analysis parameters (on the X axis) showing whether the difference in the number of detected cycles is related to the variability in the values of the calculated parameters for the audio and video data. Correlation scale on the right side of the figure: dark blue, high positive correlation; white, no correlation; dark red, high negative correlation. (**A**) Jitter group and F0; (**B**) Shimmer group.

**Figure 6:**
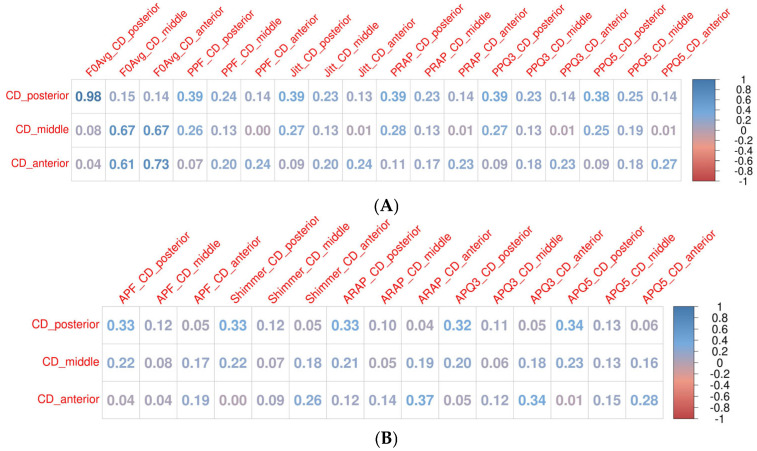
Group of patients with ≥50 cycles for the analysis. Correlations between CD index (on the Y axis) describing the compliance between cycle detection for the high-speed video (HSV) for different glottal widths and audio data (described in detail in the Section 2) and the values of the kymographic analysis parameters (on the X axis) showing whether the difference in the number of detected cycles is related to the variability in the values of the calculated parameters for the audio and video data. Correlation scale on the right side of the figure: dark blue, high positive correlation; white, no correlation; dark red, high negative correlation. (**A**) Jitter group and F0; (**B**) Shimmer group.

In this part of the study, we performed an analysis first for the whole group, which is presented in Figure 5. Secondly, we selected patients with at least 50 cycles detected for the analysis to check if the higher amount of the analyzed cycles (fifty or more) had an influence on the correlation level (Figure 6).

Our results for the whole group showed that the variability in the cycle detection was not correlated with the significant changes in the level of correlations between the values of kymographic parameters based on HSV and audio data for most of the parameters (except for F0 and Jita). To explore these dependencies, we proposed a CD index (presented in detail in the Section 2) describing the compliance between cycle detection for the high-speed video for different glottal widths and audio data and the values of the obtained parameters. Our results showed that high compliance in the cycle detection did not improve correlations between the video- and audio-based parameters (Figure 5). Moreover, the correlations were not revealed to be higher in the selected group of cases with ≥50 cycles detected (Figure 6). Comparable results were obtained for the subgroups (norm, benign, premalignant, malignant, and other) (Supplementary Figures S1–S4). In all subgroups, neither the cycle detection compliance nor the number of cycles detected exceeding 50 increased the correlation level between the parameter values for different glottal widths based on the HSV and audio data.

## 4. Discussion

High-speed videoendoscopy (HSV) is an excellent diagnostic tool for patients with dysphonia, including those with various functional and organic disorders [17,18]. Previous studies have demonstrated that HSV can serve as an auxiliary method for assessing benign and malignant laryngeal lesions [19,20]. In this paper, we demonstrate how simultaneously recorded acoustic signals enhance the accuracy and reliability of parameter calculations derived from HSV data. By cross-verifying measurements such as fundamental frequency (F0) between the two modalities, we establish a methodological advantage for combined recordings. The results suggest that simultaneous audio recordings can validate HSV-derived F0 measurements and potentially guide future methodological improvements for assessing other parameters. While this study does not directly demonstrate an enhancement in HSV accuracy, it highlights the value of integrating these modalities to improve the reliability of voice assessment. Additionally, this integration holds potential for clinical applications, such as improving diagnostic precision in evaluating vocal fold pathologies.

Although some studies suggest that HSV and acoustic parameters are closely related, such research has often been limited to small patient groups [21]. In our study, we initially examined 283 patients; however, to ensure reliable results, correlations were investigated in a subgroup of 192 patients meeting strict inclusion criteria. This cohort included individuals with functional disorders, as well as benign, premalignant, and malignant organic lesions, to explore whether the presence or type of pathology influences the correlation levels between HSV- and audio-based parameter values.

Our findings predominantly revealed low-to-moderate correlations between the HSV and audio parameters. An exception was observed in the premalignant lesions group (*n* = 15, ~10% of the cohort), where high correlations were noted for parameters describing period perturbations. Future studies on larger cohorts are needed to confirm and further investigate these results. Importantly, our study showed consistent correlation levels across subgroups, indicating that the presence or absence of pathology, as well as its type, did not significantly influence the correlations between kymographic parameters derived from HSV and those obtained from audio signals (Figure 4, Supplementary Figures S1–S4).

Our results align with those of Schlegel et al., who also reported weak correlations between HSV and audio parameters. Their study included approximately 250 patients with normophonic and functional voice disorders. A key difference, however, was our inclusion of patients with organic laryngeal lesions and the use of a different method for describing glottal function over time (GAW vs. GWW) [22]. Schlegel et al. attributed the weak correlations to the reduction in the 3D complexity of vocal fold dynamics in the GAW-calculated parameters, which they argued may obscure key laryngeal characteristics reflected in acoustic signals. They recommended exploring additional parameters for stronger correlations. Our findings support this conclusion, as we also observed low correlation levels, emphasizing the need to include 3D parameters in future analyses.

HSV offers the direct visualization of glottic pathology, unaffected by the influence of the supraglottal vocal tract. This distinction makes HSV a simpler and more reliable tool for assessing glottic pathology compared to audio signals recorded via an external microphone [11]. However, the choice of the HSV analysis method significantly affects the results. Methods accounting for the 3D complexity of vocal fold motion, such as those proposed by Patel et al., could improve assessments of vocal fold function. Their custom laser endoscope enabled precise measurements of lateral and vertical vibratory motion across anterior, middle, and posterior cross-sections of the vocal folds, highlighting the importance of assessing vertical movements of the superior vocal fold surface. However, their study did not include audio data analysis, leaving room for future research to explore the relationships between 3D HSV parameters and acoustic data [23].

Simultaneous recording of HSV and audio signals is recommended by some researchers to ensure the accuracy of HSV analysis, particularly for verifying F0 values [11]. F0 is a stable parameter, consistent across different hardware and software, even under suboptimal conditions [24,25]. In our study, F0 correlations were very high (~0.97) across all glottal widths and audio data, validating the accuracy of our HSV recordings and analyses. These findings are consistent with our preliminary study on flexible endoscopes for HSV, where F0 values were comparable across rigid and flexible endoscopes and corresponding audio data [15].

High correlations were also observed for mean Jitter (Jita) in our study. This likely reflects the mathematical relationship between Jita and F0, as Jita is calculated by dividing Jitter by the mean fundamental frequency (F0). Empirically, Jita tends to exhibit stronger correlations with F0 than Jitter, which accounts for relative variability and may better align with the logarithmic nature of human pitch perception. The relationship between Jitter parameters and F0 is complex, combining mathematical dependency with physiological and perceptual factors. Jitter, in order to potentially account for the reduction in its perceptual dependency and obtain as good as possible independence from the average F0, is mathematically defined as the ratio of the difference of the periods to their average value. Jita, on the other hand, is mathematically defined as the ratio of Jitter to the average fundamental period, that is, as the product of Jitter and fundamental frequency. Assuming, therefore, that the definition of Jitter does indeed eliminate the dependence on F0 as much as possible, Jita will be directly linearly dependent on F0 through the fundamental mathematical relationship. Our results demonstrate stronger correlations between Jita and F0 compared to Jitter, consistent with the hypothesis that Jitter better reflects pitch variability across a range of frequencies. This distinction is critical for interpreting Jitter metrics in both research and clinical contexts, as it underscores the importance of considering F0 when analyzing and comparing these parameters. Jita’s stability across glottal widths makes it a promising parameter for cases with challenging glottis visualization. Previous studies have demonstrated Jita’s diagnostic significance in differentiating healthy individuals from those with organic pathologies, with high AUC values when used alone or in models distinguishing lesion types [19]. The middle and posterior glottal cross-sections are particularly reliable for Jita analysis, even when the entire glottal gap is not visualized [26].

Cycle detection inconsistencies are a known challenge in comparing HSV and audio parameters, as noted by Bielamowicz [27]. In our study, the number of detected cycles was analyzed to assess its impact on correlations. Complete compliance in cycle detection between HSV and audio data was observed in only 20% of patients. This percentage increased to 50% when at least one HSV glottal width was considered and further to ~50% for patients with a variability coefficient ≤ 2% across the three glottal widths and audio data.

Organic lesions can impact local vocal fold structures, which may explain discrepancies in cycle detection across different glottal regions. The worst compliance was observed in patients with benign lesions, particularly those with large polyps or Reinke’s edema, as these conditions cause asymmetry, increased mucosal wave propagation, and amplitude variations, complicating software-based cycle detection. Despite these challenges, HSV proved more effective than LVS for assessing severe Reinke’s edema, especially in cases where LVS failed due to strobe synchronization issues [28].

Unlike previous studies that focused on smaller or more specific patient groups, our study examined a large cohort of 192 participants encompassing diverse vocal pathologies. This breadth allows for more robust insights into the generalizability and reliability of HSV and acoustic parameters across clinical contexts. This study showed the validity of performing acoustic analysis during HSV endoscopy to check the correctness of quantifying kymographic parameters. An important novel aspect of this study is the focused analysis of premalignant lesions. Our findings reveal that this subgroup exhibits high correlations for certain parameters, such as Jitter-related metrics, which have not been extensively explored in prior research. Our study introduces the evaluation of cycle detection variability as a novel methodological approach. By analyzing the impact of variability on parameter correlations, we provide insights into the reliability of HSV and acoustic measurements, addressing a critical gap in the literature regarding software performance and its implications for clinical use.

Using an ICC model evaluating consistency, our findings confirm that the correlation between HSV and audio-based parameters remains robust, supporting the reliability of both modalities while accounting for possible systematic differences in measurement techniques. For example, fundamental frequency (F0) exhibited both high correlation (r = 0.97) and excellent ICC values, confirming strong concordance between the two measurement methods. Conversely, moderate ICC values for parameters such as Shimmer highlight the need for further research to better understand these discrepancies.

This study focused on kymographic parameters characterizing the period and amplitude perturbations for their dual applicability to HSV and acoustic analyses. Other parameters, such as asymmetry or open quotient, and alternative methodologies like phonovibrograms, were not included. Future research should investigate additional HSV-derived parameters and their correlations with audio data, particularly in characterizing specific lesions of the glottis. Reducing the number of analyzed parameters may streamline the diagnostic process, enhancing its clinical utility. Identifying the most critical parameters for glottic pathology evaluation and post-phonosurgical monitoring is a key priority. Moreover, the clinical utility of the parameters obtained from the audio data recorded simultaneously with the HSV should be assessed both in the diagnostic process and follow-up assessment. By combining correlation results from this study, we can identify redundant parameters and eliminate collinearity in future analyses. This approach allows for a more precise evaluation of the unique contributions of audio data in complementing HSV-based assessments.

## 5. Conclusions

Kymographic parameters derived from HSV showed predominantly low-to-moderate correlations with simultaneously recorded audio data, with the notable exception of high correlations for F0 and mean Jitter (Jita). While not all parameters describing the vocal fold oscillations could include the information of 3D complex structure, there are a lot of others in which the integration of 3D imaging methods provides a better capture of the complex vibratory dynamics of vocal folds. Additionally, HSV remains a valuable diagnostic tool, particularly in cases where LVS is insufficient, offering precise insights into vocal fold function regardless of pathology.

Cycle detection consistency and the number of detected cycles did not influence correlation levels, suggesting the additional influence of the upper part of the vocal tract on audio signals and the loss of 3D vocal fold dynamics in HSV analysis rather than software limitations. While F0 values confirmed the accuracy of HSV recordings, further research is needed to integrate 3D analyses and identify parameters that reliably capture the complexities of phonation. Until then, both HSV and audio-based parameters should be used to provide a comprehensive functional assessment of the glottis.

## Figures and Tables

**Table 1:** The number of subjects with a particular diagnosis.

Diagnosis	Number of Subjects
Normophonic	36 (18.7%)
Benign	71 (37%)
Precancerous	15 (7.8%)
Malignant	29 (15.1%)
Other	41 (21.4%)

**Table 2:** Presentation of both Pearson correlation and ICC (3,1) values, providing additional insight into the agreement between the HSV-derived and acoustic parameters.

Parameter	Pearson Correlation (r)	ICC (3,1) (95% CI)	Agreement Level
Fundamental Freq. (F0)	0.97	0.91 (0.88–0.94)	Excellent
Mean Jitter (Jita)	0.85	0.76 (0.73–0.80)	Good
Shimmer	0.68	0.62 (0.58–0.66)	Moderate

## Data Availability

In the Supplementary Materials.

## Supplementary Materials

The following supporting information can be downloaded at https://www.mdpi.com/article/10.3390/biomedicines13020511/s1. Table S1. Correlations between kymographic parameters based on the high-speed videoendoscopy (HSV) including different glottal widths (anterior, middle, and posterior) and parameters based on simultaneously recorded audio signals—subjects with benign lesions; Table S2. Correlations between kymographic parameters based on the high-speed videoendoscopy (HSV) including different glottal widths (anterior, middle, and posterior) and parameters based on simultaneously recorded audio signal—subjects with premalignant lesions; Table S3. Correlations between kymographic parameters based on the high-speed videoendoscopy (HSV) including different glottal widths (anterior, middle, and posterior) and parameters based on simultaneously recorded audio signals—subjects with malignant lesions; Supplementary Table S4. Correlations between kymographic parameters based on the high-speed (HSV) including different glottal widths (anterior, middle, and posterior) and parameters based on simultaneously recorded audio signals—subjects with functional disorders; Table S5. Correlations between kymographic parameters based on the high-speed videoendoscopy (HSV) including different glottal widths (anterior, middle, and posterior) and parameters based on simultaneously recorded audio signals—whole group; Figure S1. Subjects with benign lesions. Correlations between CD index (on the Y axis) describing the compliance between cycle detection for the high-speed video (HSV) for different glottal widths and audio data (described in detail in the Section 2) and the values of the kymographic analysis parameters (on the X axis) showing whether the difference in the number of detected cycles is related to the variability in the values of the calculated parameters for the audio and video data. Correlation scale on the right side of the figure: dark blue, high positive correlation; white, no correlation; dark red, high negative correlation. A—Jitter group and F0; B—Shimmer group; Figure S2. Subjects with premalignant lesions. Correlations between CD index (on the Y axis) describing the compliance between cycle detection for the high-speed video (HSV) for different glottal widths and audio data (described in detail in the Section 2) and the values of the kymographic analysis parameters (on the X axis) showing whether the difference in the number of detected cycles is related to the variability in the values of the calculated parameters for the audio and video data. Correlation scale on the right side of the figure: dark blue, high positive correlation; white, no correlation; dark red, high negative correlation. A—Jitter group and F0; B—Shimmer group; Figure S3. Subjects with malignant lesions. Correlations between CD index (on the Y axis) describing the compliance between cycle detection for the high-speed video (HSV) for different glottal widths and audio data (described in detail in the Section 2) and the values of the kymographic analysis parameters (on the X axis) showing whether the difference in the number of detected cycles is related to the variability in the values of the calculated parameters for the audio and video data. Correlation scale on the right side of the figure: dark blue, high positive correlation; white, no correlation; dark red, high negative correlation. A—Jitter group and F0; B—Shimmer group; Supplementary Figure S4. Subjects with functional disorders. Correlations between CD index (on the Y axis) describing the compliance between cycle detection for the high-speed video (HSV) for different glottal widths and audio data (described in detail in the Section 2) and the values of the kymographic analysis parameters (on the X axis) showing whether the difference in the number of detected cycles is related to the variability in the values of the calculated parameters for the audio and video data. Correlation scale on the right side of the figure: dark blue, high positive correlation; white, no correlation; dark red, high negative correlation. A—Jitter group and F0; B—Shimmer group.
