# Supplementary figures and images for: Assessment of the Interdependencies Between High-Speed Videoendoscopy and Simultaneously Recorded Audio Data in Various Glottal Pathologies
Source: Biomedicines. 2025 Feb 18;13(2):511. doi: 10.3390/biomedicines13020511 (PMC11852736; doi:10.3390/biomedicines13020511)

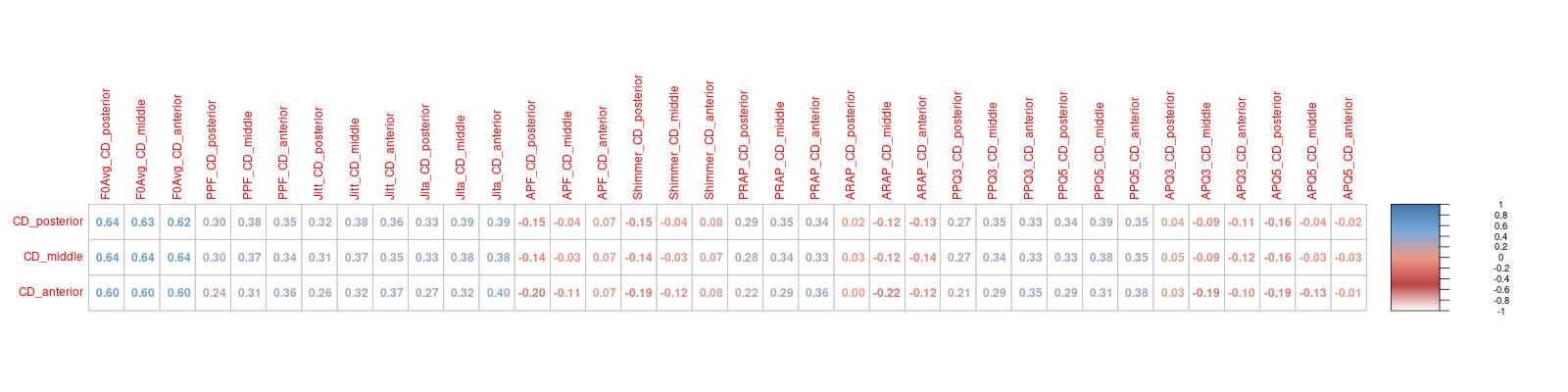

Supplement: Supplementary file 1 [file biomedicines-13-00511-s001.zip › Figure S1.jpg]

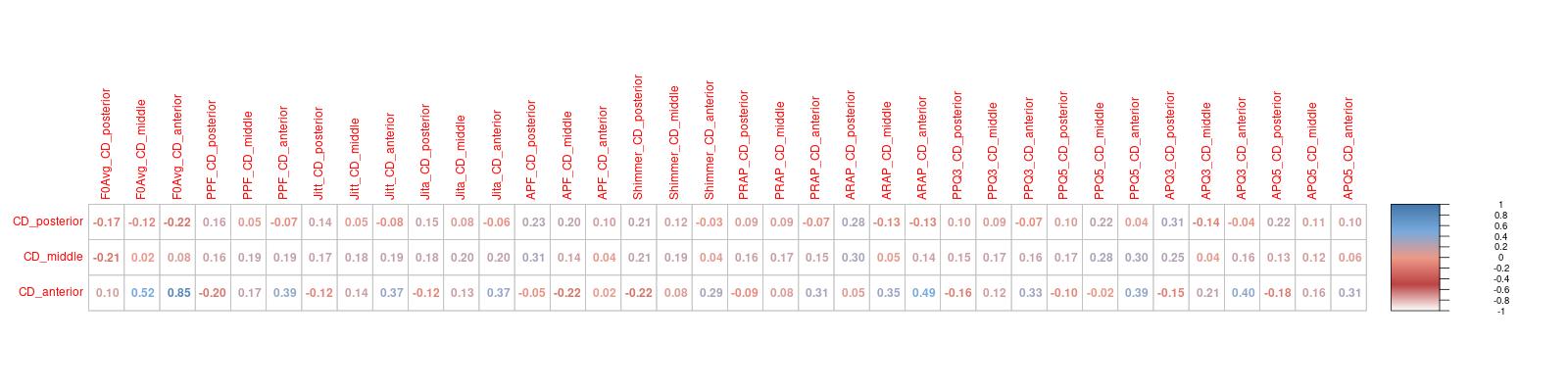

Supplement: Supplementary file 1 [file biomedicines-13-00511-s001.zip › Figure S2.jpg]

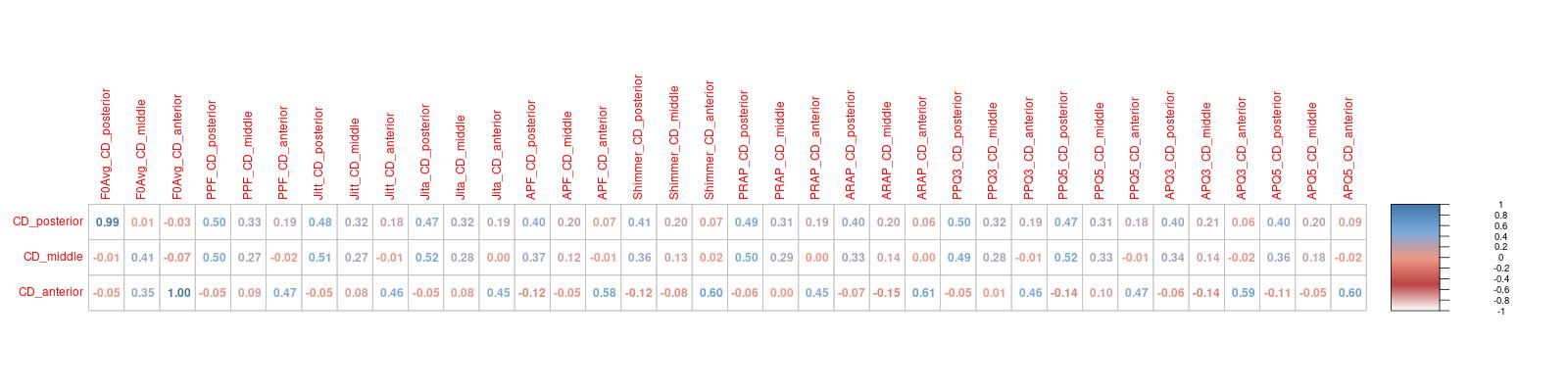

Supplement: Supplementary file 1 [file biomedicines-13-00511-s001.zip › Figure S3.jpg]

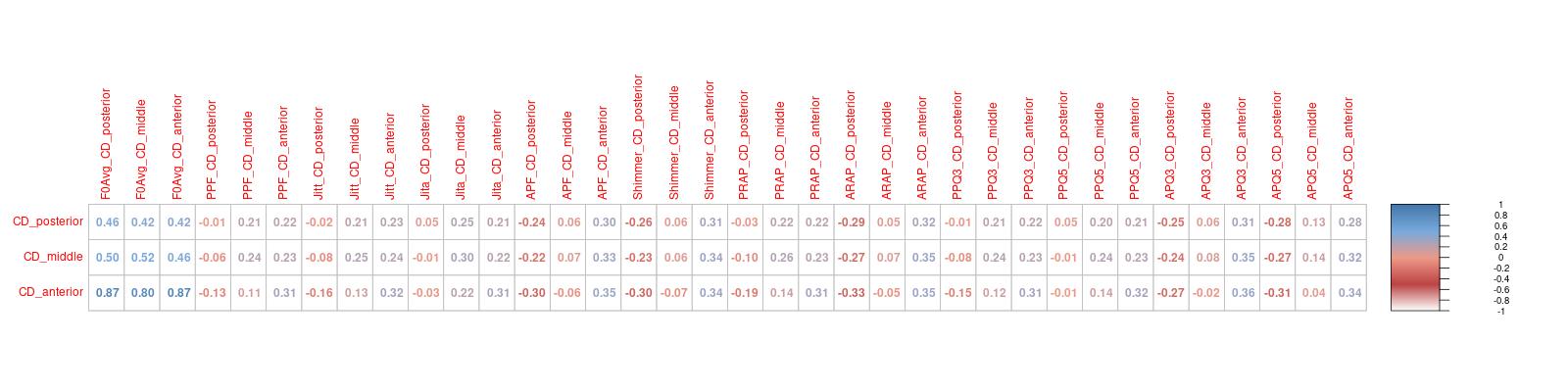

Supplement: Supplementary file 1 [file biomedicines-13-00511-s001.zip › Figure S4.jpg]
